# Modeling of moral decisions with deep learning

**DOI:** 10.1186/s42492-020-00063-9

**Published:** 2020-11-20

**Authors:** Christopher Wiedeman, Ge Wang, Uwe Kruger

**Affiliations:** 1 Department of Electrical and Computer Systems Engineering, Rensselaer Polytechnic Institute, Troy, NY USA; 2 Department of Biomedical Engineering, Rensselaer Polytechnic Institute, Troy, NY USA

**Keywords:** Artificial intelligence, Deep learning, Bayesian method, Moral machine experiment

## Abstract

One example of an artificial intelligence ethical dilemma is the autonomous vehicle situation presented by Massachusetts Institute of Technology researchers in the *Moral Machine Experiment*. To solve such dilemmas, the MIT researchers used a classic statistical method known as the hierarchical Bayesian (HB) model. This paper builds upon previous work for modeling moral decision making, applies a deep learning method to learn human ethics in this context, and compares it to the HB approach. These methods were tested to predict moral decisions of simulated populations of *Moral Machine* participants. Overall, test results indicate that deep neural networks can be effective in learning the group morality of a population through observation, and outperform the Bayesian model in the cases of model mismatches.

## Introduction

With the rapid development toward automation, future reliance on artificial intelligence (AI) for everyday tasks is clear. Often embedded within these tasks are small moral decisions: for example, is violating a minor traffic law justified when it saves the time of others? While humans take these small ethical decisions for granted, society must properly equip AI products with moral compasses if we are to entrust machines even with small daily tasks. Furthermore, confidence in an AI’s ability to make sensible moral decisions is key to winning public acceptance of such systems.

Public acceptance of AI as responsible moral agents is one of the greatest obstacles facing automation and machine learning. Bigman and Gray [1] highlights that people have shown distinct aversion to entrusting machines with ethical decisions in multiple studies, despite the fact that AI has demonstrated superior judgement to humans in certain domains. Other research and surveys indicate that a person’s previous exposure to machine-made decisions plays a crucial role in their confidence in ethical AI [2]. Formulating and demonstrating an easily applicable approach to programming moral agents is the first step in earning public trust in this domain.

Incorporating moral sensibility into machines remains challenging, as it is difficult to derive a quantitative model for objectively determining moral decisions. Current research in AI moral decision making often theorizes abstract and general approaches to training moral agents [3, 4] For example, Shaw et al. [4] proposes a machine learning framework where a group of statistically trained models determine a moral action based on each individual model’s decision, and the confidence each model has in the morality of other models [4]. Still, reducing complex moral scenarios to a form that a framework can easily digest is obtuse.

As with many problems, researchers can find inspiration in human cognitive abilities, including moral determination. English philosopher Jeremy Bentham theorized that individuals choose actions that yield the greatest social utility when faced with ethical dilemmas [5]. Research in universal moral grammar has supported this notion, additionally noting that the moral value of a decision also depends on the context and actions an agent must take within that decision, and not just the net result [6]. As such, it may be possible to model ethical decisions based upon the social utility of each option within a decision. In this paper, we investigate a deep learning based moral decision model, taking a hypothetical autonomous vehicle dilemma as an example.

### The moral machine experiment

**Fig. 1:**
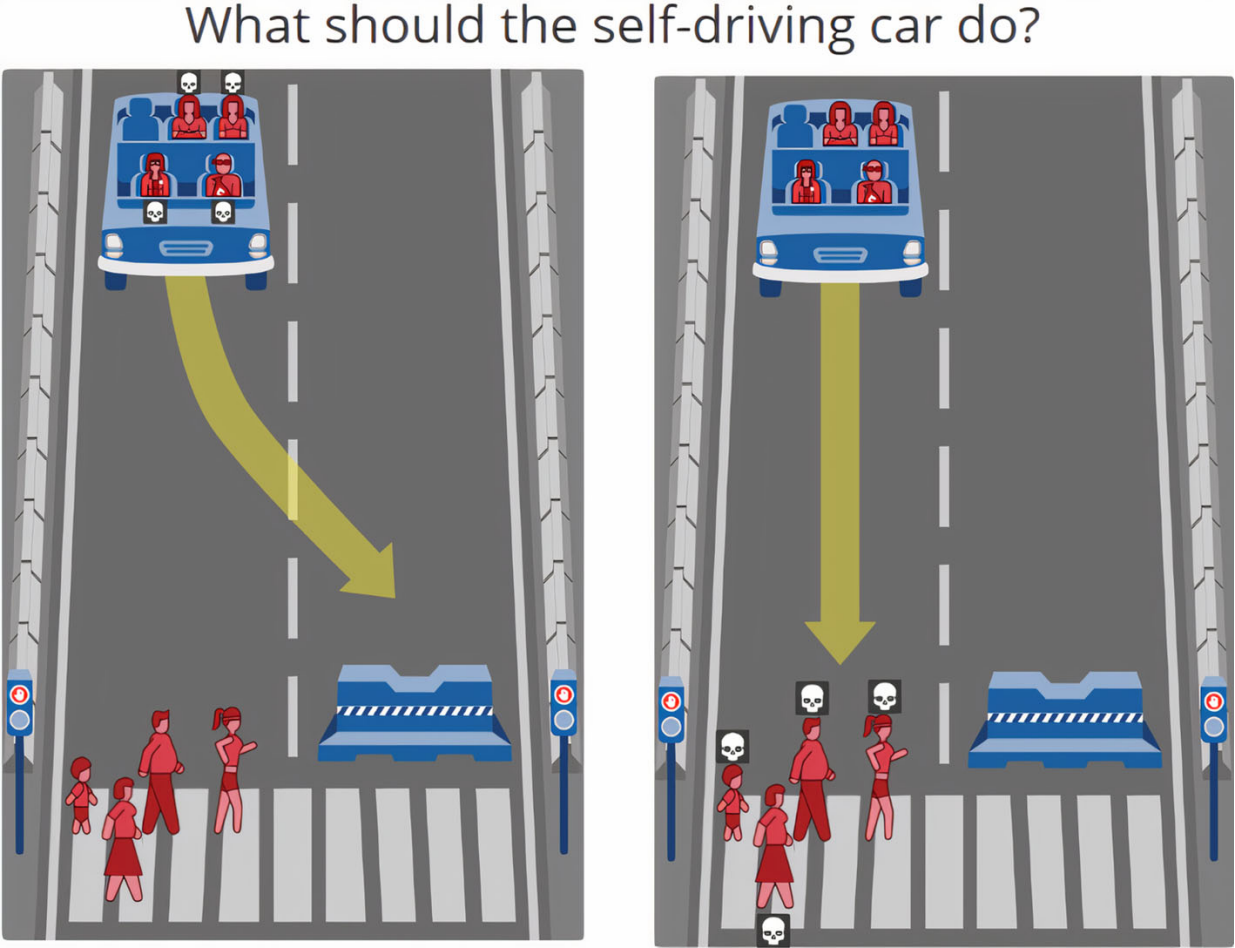
Moral Machine Example: Example screenshot of a scenario from a Moral Machine scenario [8]. The participant must decide the more ethical course of action for the self-driving vehicle: swerving would kill all vehicle passengers (right), while maintaining the course would result in the death of all pedestrians (left) [8]

One scenario relevant to ethical modeling with social utility is the imminent crash of a self-driving vehicle: in this hypothetical situation, an autonomous vehicle with a catastrophic brake failure must decide between killing one of two distinct groups of people. This scenario is a suitable starting point for discussing ethical AI decision making, as it has been investigated extensively in the *Moral Machine Experiment* [7]. This experiment surveyed thousands of people worldwide for their preferences in autonomous vehicle ethical dilemmas [7]. In any given instance, a participant would be presented with an unwinnable scenario in which only one of two groups of people could survive (see Fig. 1). The survey aggregated answers based on region and evaluated the moral values that societies generally place on different abstract dimensions, such as age, social status, law adherence, and gender. Observing the data and attempting to transfer scenarios into comparative costs based on abstract values is the first step in creating a model that can ethically make these decisions.

### Hierarchical Bayesian modeling for moral decision making

**Fig. 2:**
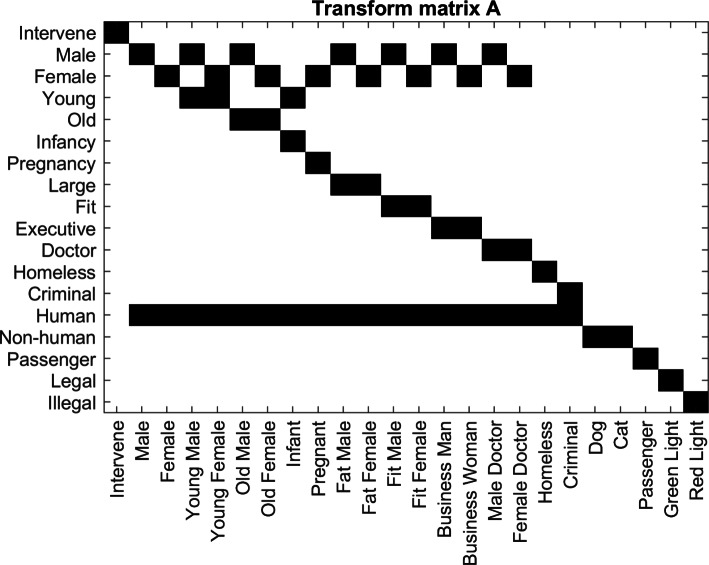
Feature Transform: Binary transformation matrix *A* used in Kim, et al., which converts a set of character traits *θ* into quantifiable features [9]

Before describing a deep learning model, it is necessary to both credit and summarize the work by Kim, et al. in *A Computational Model of Commonsense Moral Decision Making* [9]. This paper observes the same scenario from the Moral Machine Experiment, and models human moral decisions as a random process based on the perceived social utilities between options in a scenario. Each autonomous vehicle scenario contains two options (*y*=0,1), and option *y* can be characterized by vector *θ*_*y*_. The characters within *θ*_*y*_ hold various features (such as male, human, doctor, young, etc.). The total features of *θ*_*y*_ can be found after applying a linear transformation *λ*=*F*(*θ*)=*Aθ*, where *λ* is the sum of features in *θ*. Figure 2 shows the linear transform *A* used in Kim, et al. [9].

Kim, et al. models human decisions in these scenarios as a comparison of perceived social utilities. The social utility *u* of option *i* is calculated as follows:

1 $$\begin{array}{@{}rcl@{}} u\left(\theta_{i}\right) = w^{\top} F\left(\theta_{i}\right)  \end{array} $$

where a vector *w* is an individual’s set of moral abstract weights for each feature [9]. For each scenario containing two options, *i*=0,1 (non-intervention and intervention respectively), the probability that the individual will choose the intervention function is modeled as [9]

2 $$\begin{array}{@{}rcl@{}} P(Y=1|\theta_{0,1}) = \frac{1}{1+e^{{-(u(\theta_{1})-u(\theta_{0}))}}} \end{array} $$

At the heart of this modeling, the *w* vector for a participant quantifies the abstract moral values that the individual holds for different features (for example, the value of the 4th vector element corresponds to the social value that individual places on youth). Furthermore, Kim, et. al assume that the distribution of these moral values for a culture can be characterized as a multivariate normal distribution, where the mean *w*^*g*^ represents the group average, and the covariance matrix *Σ*^*g*^ represent in-group variances and co-dependence of values (for example, value for pregnancy are correlated with value for infancy).

3 $$\begin{array}{@{}rcl@{}} w_{i} \sim\ Normal\left(w^{g}, \Sigma^{g}\right) \end{array} $$

Assuming this underlying model, Kim, et. al proposed a Hierarchical Bayesian model, which observes participants’ decisions in the Moral Machine experiment, and predicts individual decisions by inferring underlying moral value set *w*_*i*_ for each individual.

### Creating a model without assuming an underlying normal distribution

The Hierarchical Bayesian model proves valuable in predicting decisions in the Moral Machine experiment [9]. Its efficacy, however, relies upon the assumption that the modeled abstract values are normally distributed. Indeed, many moral values result from a linear summation of other values, and thus will tend toward a normal distribution by the Central Limit Theorem. It is also possible, however, that other moral values are a more complex, non-linear function of other factors. Thus, it may not always be safe to assume an underlying normal distribution of moral values pertaining to a specific ethical dilemma.

In this paper, we propose the use of a deep neural network for predicting individual moral outcomes. While deep neural networks generally require sufficient amounts of training data, they do not require any prior assumptions regarding the decision process or population distributions of moral principles. Rather, a neural network implicitly learns these aspects through observation. In the following work, we train a deep neural network to predict individual moral decisions in the autonomous vehicle scenario, and compare its performance to that of a Hierarchical Bayesian model. We simulate participant decisions by maintaining the same decision-making process from Kim, et al. [9], but vary the underlying distribution of *w*, representing different possible distributions of moral principles.

## Methods

Three models are considered for predicting individual moral decisions from Moral Machine scenarios: a deep learning model, a Bayesian model in which an underlying distribution was assumed, and a likelihood model where no distribution assumptions are made. These models were tested with simulated Moral Machine survey data, which were generated with various underlying distributions of moral values.

### Participant simulation

**Fig. 3:**
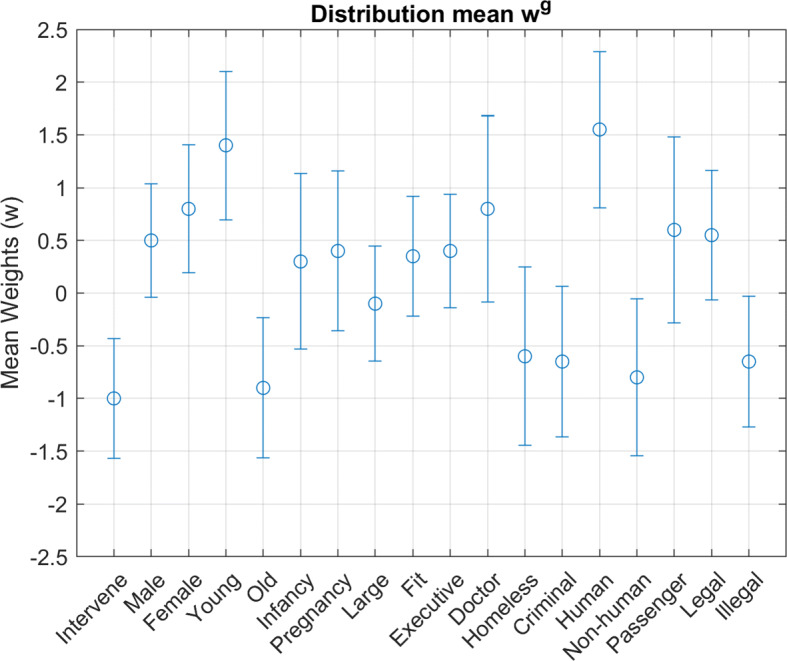
Moral Principle Mean: Mean value for all underlying distributions of the moral principle vector *w*

**Fig. 4:**
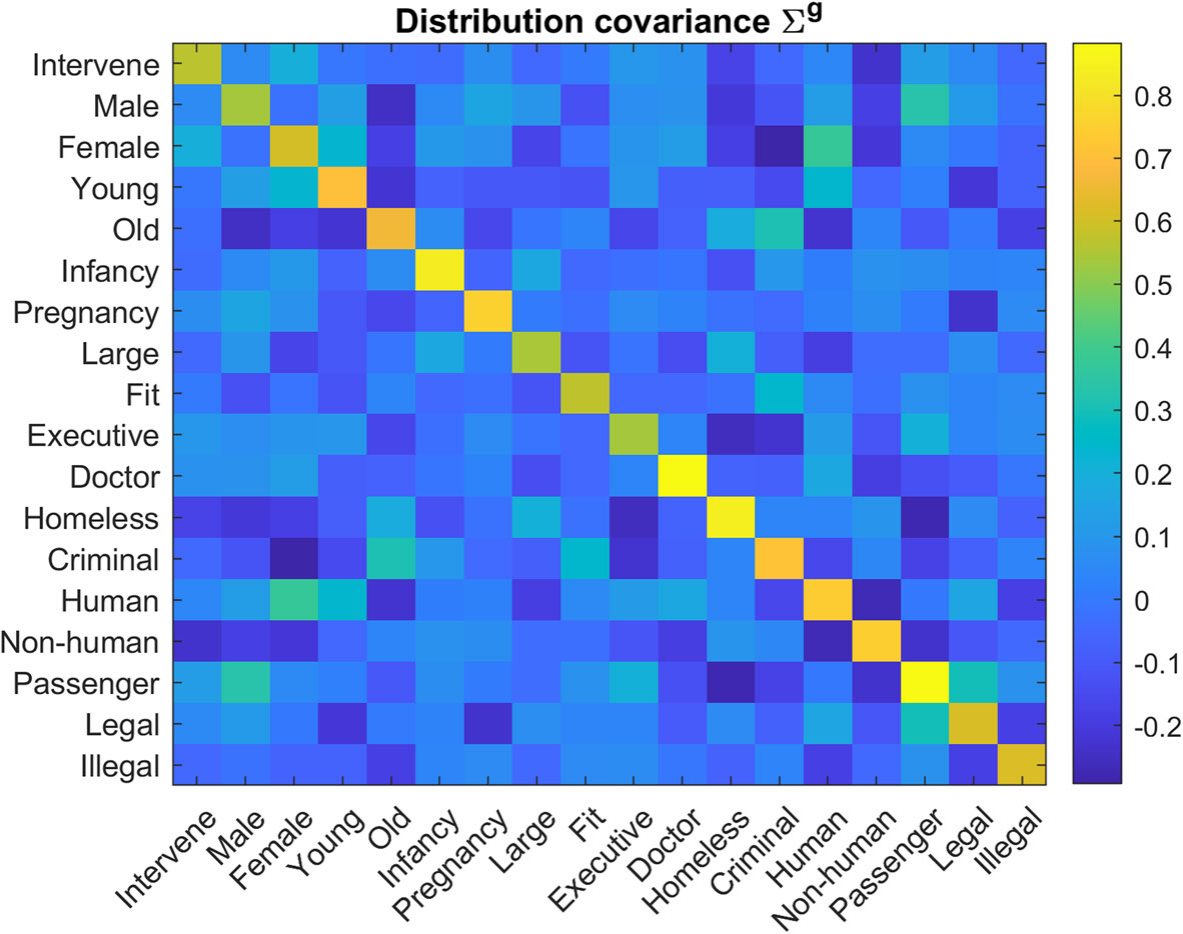
Moral Principle Covariance: Covariance matrix for all underlying distributions of the moral principle vector *w*

Each virtual participant is characterized by their personal moral vector *w*, sampled from a population’s multivariate distribution. To create a normally distributed dataset, participants were i.i.d. sampled per Eq. 3, where group mean *w*^*g*^ and covariance *Σ*^*g*^ are specified in Figs. 3 and 4 respectively. These parameters were selected roughly based upon the inferred distribution parameters for Danish participants in Kim, et al. [9].

Then, five generalized Gaussian multivariate distributions were created, with the probability density function (PDF) *f*(*x*):

4 $$\begin{array}{@{}rcl@{}} {}{f(x)} &=& \frac{\phi(y)}{\alpha-\kappa\left(x-\xi\right)}; \end{array} $$

$$\begin{array}{@{}rcl@{}} y &=& -\frac{ln\left(1-\frac{\kappa\left(x-\xi\right)}{\alpha}\right)}{\kappa}  \end{array} $$

where *ϕ* is the standard normal PDF function, *κ*,*α*, and *ξ* are the shape parameter, scale parameter, and median respectively. The mean and variance for this distribution are as follows [10]:

5 $$\begin{array}{@{}rcl@{}}  \mu &=& \xi - \frac{\alpha}{\kappa}\left(e^{\kappa^{2}/2-1}\right) \end{array} $$

6 $$\begin{array}{@{}rcl@{}} \sigma^{2} &=& \frac{\alpha^{2}}{\kappa^{2}}e^{\kappa^{2}}\left(e^{\kappa^{2}}-1\right) \end{array} $$

Each of these distributions was sampled by first sampling the previous normal distribution. Then, each element *i* in sample *w* was transformed to a target non-Gaussian distribution with the equation:

7 $$\begin{array}{@{}rcl@{}} \hat{w_{i}} &=& \frac{\alpha_{i}}{\kappa_{i}}\left(1-e^{-\kappa_{i} \frac{w_{i}-\mu_{i}}{\sigma_{i}}}+ \xi_{i}\right) \end{array} $$

*μ*_*i*_ and *σ*_*i*_ are the marginal mean and standard deviation respectively for the moral component value *i*. *κ* values for a target distribution were generated (see Table 1). The scale parameter *α*_*i*_ and median *ξ*_*i*_ were then calculated based upon *κ*_*i*_ with the following equations, ensuring that the mean and covariance of the distribution are preserved during the transformation:

8 $$\begin{array}{@{}rcl@{}} \alpha_{i} &=& \frac{\mid \kappa_{i} \sigma_{i} \mid}{\sqrt{e^{\kappa^{2}}\left(e^{\kappa^{2}}-1\right)}} \end{array} $$

**Table 1:** *κ* values generated for five non-gaussian distributions

∣*k*∣=	0.5	1	1.5	2	2.5
Intervene	0.77	1.5	-0.37	-0.69	2.61
Male	0.25	-1.66	-1.28	1.24	2.05
Female	-0.8	0.52	0	-0.8	1.59
Young	0.34	-0.76	-1.35	3.79	-4.59
Old	0.01	-0.1	1.99	-1.84	4.19
Infancy	-0.15	-0.87	-1.84	-1.05	1.1
Pregnancy	-0.09	-0.23	-2.31	3.45	-2.68
Large	0.51	-1.95	-2.86	3.52	-4.36
Fit	0.59	1.01	-0.86	1.57	0.88
Executive	0.76	1.05	-1.82	-1.31	4.55
Doctor	-0.53	-0.2	1.17	2.86	1.2
Homeless	0.88	-1.33	2.82	2.4	1.58
Criminal	-0.34	0.9	2.47	-2.14	4.11
Human	0.66	-0.88	-1.78	2.82	1.7
Non-Human	0.59	-0.42	-1.34	1.43	-3.88
Passenger	0.84	-1.95	0.05	1.07	1.26
Legal	-0.07	-1.55	-1.38	0.73	-1.67
Illegal	0.59	-1.31	1.17	-2.9	-1.9

9 $$\begin{array}{@{}rcl@{}} \xi_{i} &=& \mu_{i} + \frac{\alpha_{i}}{\kappa_{i}}\left(e^{\frac{\kappa_{i}^{2}}{2}}-1\right) \end{array} $$

**Fig. 5:**
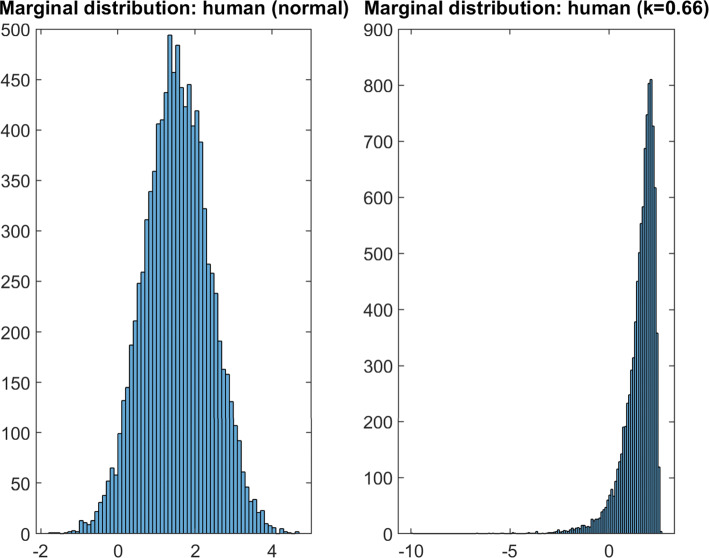
Distribution Transform: Histograms of marginal distributions of the ’Human’ value for the normal dataset (left) and one of the transformed datasets (right)

The *κ* values for the five non-Gaussian distributions were randomly generated such that each distribution displayed a different degree of skew, as evident by each distribution’s approximate average *κ* magnitude. Each distribution was i.i.d. sampled to create 3,000 participant datasets. For each dataset, 1,000 of these samples was set aside as a test set. An example of the transform’s effect on a marginal distribution is shown in Fig. 5.

### Scenario and decision simulation

Similar to the actual Moral Machine experiment, each of the simulated participants in each group was given thirteen moral machine scenarios. The parameters *θ*_0_,*θ*_1_ of each scenario were randomly generated. Each option randomly has 0-5 people present, with a 75% chance that each option in one scenario contains an equal number of people (this is done to avoid trivial comparisons, such as 2 people vs 12, and having all decisions be completely dominated by the total number of people in either option). Certain values were preset or selected as a binary to ensure that scenarios were feasible within the Moral Machine framework (for example, the ’Intervene’ value was always 1 for *θ*_1_ and 0 for *θ*_0_). It should be noted that the parameter generation of each scenario is largely random, while survey questions in the Moral Machine experiment are mostly targeted towards isolating a single factor (gender, social status, etc.) [7].

The decisions from each participant were modeled as the random process described with Eqs. 1 and 2, and as outlined in Kim, et al. [9]. In separate tests, decisions were simulated with a deterministic version of Eq. 2, in which the maximum likelihood decision was always chosen (discussed in “Results and discussions” section).

### Model creation and testing

A hierarchical Bayesian (HB), maximum likelihood (ML), and deep learning (DL) model for predicting moral decisions were created and tested with representative participant distributions. The key details are described in the following.

#### Hierarchical Bayesian model

For participant *i*, who handled scenarios $\Theta _{i} = \left [\Theta _{i}^{1},... \Theta _{i}^{N}\right ]$ with decisions $Y_{i} = \left [y_{i}^{1},... y_{i}^{N}\right ]$, the HB model maximized the posterior probability of *w*_*i*_, and used this estimate to predict other scenario decisions. The Bayesian model is based on the following equations from Kim et al. [9]:

10 $$\begin{array}{@{}rcl@{}} P\left(w_{i}, w^{g}, \Sigma^{g}\right) &\propto P\left(\Theta_{i}, Y_{i}|w_{i}\right) P\left(w_{i}|w^{g}, \Sigma^{g}\right) \\ &P\left(w^{g}\right)P\left(\Sigma^{g}\right)  \end{array} $$

with likelihood:

11 $$ \begin{aligned} P\left(\Theta_{i}, Y_{i}|w_{i}\right) = \prod\limits_{k=1}^{N} P\left(y_{i}^{k} = 1 |\Theta_{i}^{k}\right)^{y_{i}^{k}} \left(P\left(y_{i}^{k} = 0 | \Theta_{i}^{k}\right)^{\left(1-y_{i}^{k}\right)}\right) \end{aligned}  $$

This model was given the exact *w*^*g*^ and *Σ*^*g*^, but always assumed an underlying normal distribution of *w*; i.e., *P*(*w*^*g*^)=*P*(*Σ*^*g*^)=1,*P*(*w*_*i*_|*w*^*g*^,*Σ*^*g*^)∝*ϕ*(*w*_*i*_) where *ϕ* is the normal multivariate pdf with mean and covariance *w*^*g*^,*Σ*^*g*^. Thus, this model represents a best scenario where a Bayesian model infers the distribution hyperparameters optimally, but assumes a normal underlying distribution, potentially mismatching the true underlying distribution.

#### Maximum likelihood model

The ML model is similar to the HB, but does not assume any information regarding the underlying distribution. Thus, for each individual, ML estimates for *w*_*i*_ by maximizing the likelihood in Eq. 10.

#### Deep learning model

**Fig. 6:**
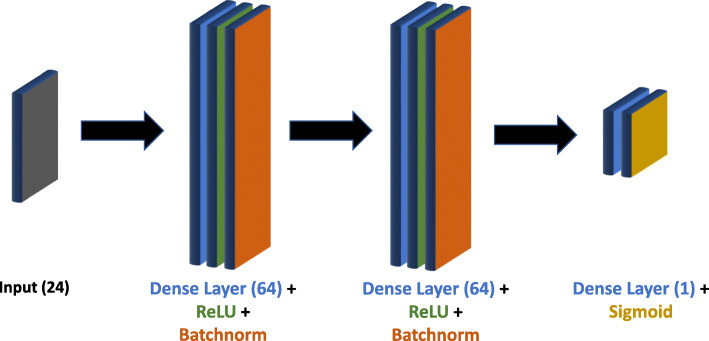
Neural Network Architecture: Network architecture for the DL moral decision model. Batchnorm signifies a batch normalization layer

Unlike the other two models, the DL model does not explicitly estimate any moral principle vector *w*. Rather, it directly predicts a decision *y* from a vector of scenario parameters *θ*. Scenario *i* is input into the network as a length 24 vector reflecting $\Theta ^{i}_{1}$ and $\Theta ^{i}_{0}$. The model architecture consists of a sequence of densely connected layers with batch normalization and ReLU or sigmoid activation (Fig. 6). Binary cross entropy is used as a loss function. A learning rate of 5*e*^−4^ with a decay rate of 0.1 was used for training. For each distribution, the network was trained with sample sizes of 25, 50, 200, 500, 1,000, and 2,000 participants (per simulated participant, 8 questions were used for training, and 5 were used for validation). Finally, testing was conducted on the test set for each distribution, in which predictions from five test questions per simulated participant were evaluated. Initially, the neural network underwent an ’individual fine-tune’ in which the network parameters were briefly fine-tuned with eight questions worth of individual-specific data, but this practice was discarded, as it was found to have no significant effect on performance (discussed in “Results and discussions” section).

### Generation/Testing on generalized data

**Fig. 7:**
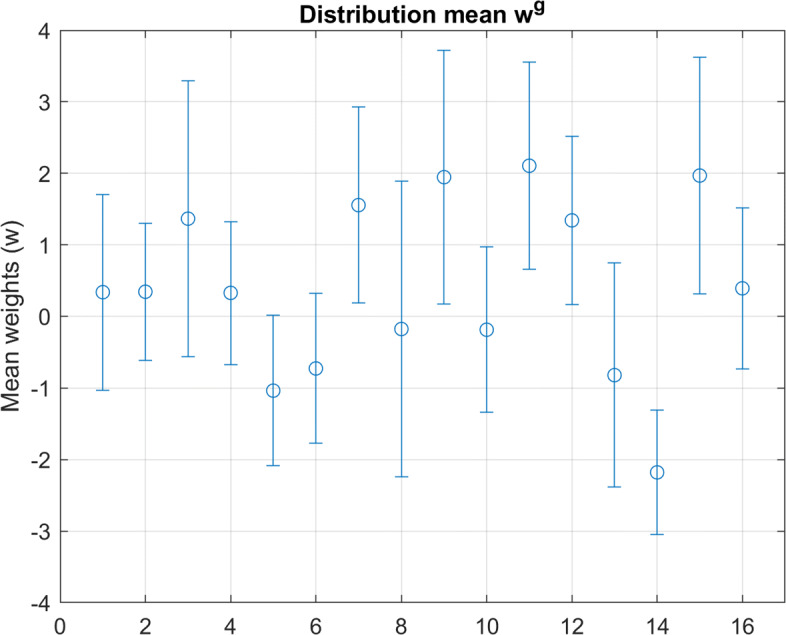
Generated Distribution Mean: Mean values of the principle vector *w* used to synthesize more general data

**Fig. 8:**
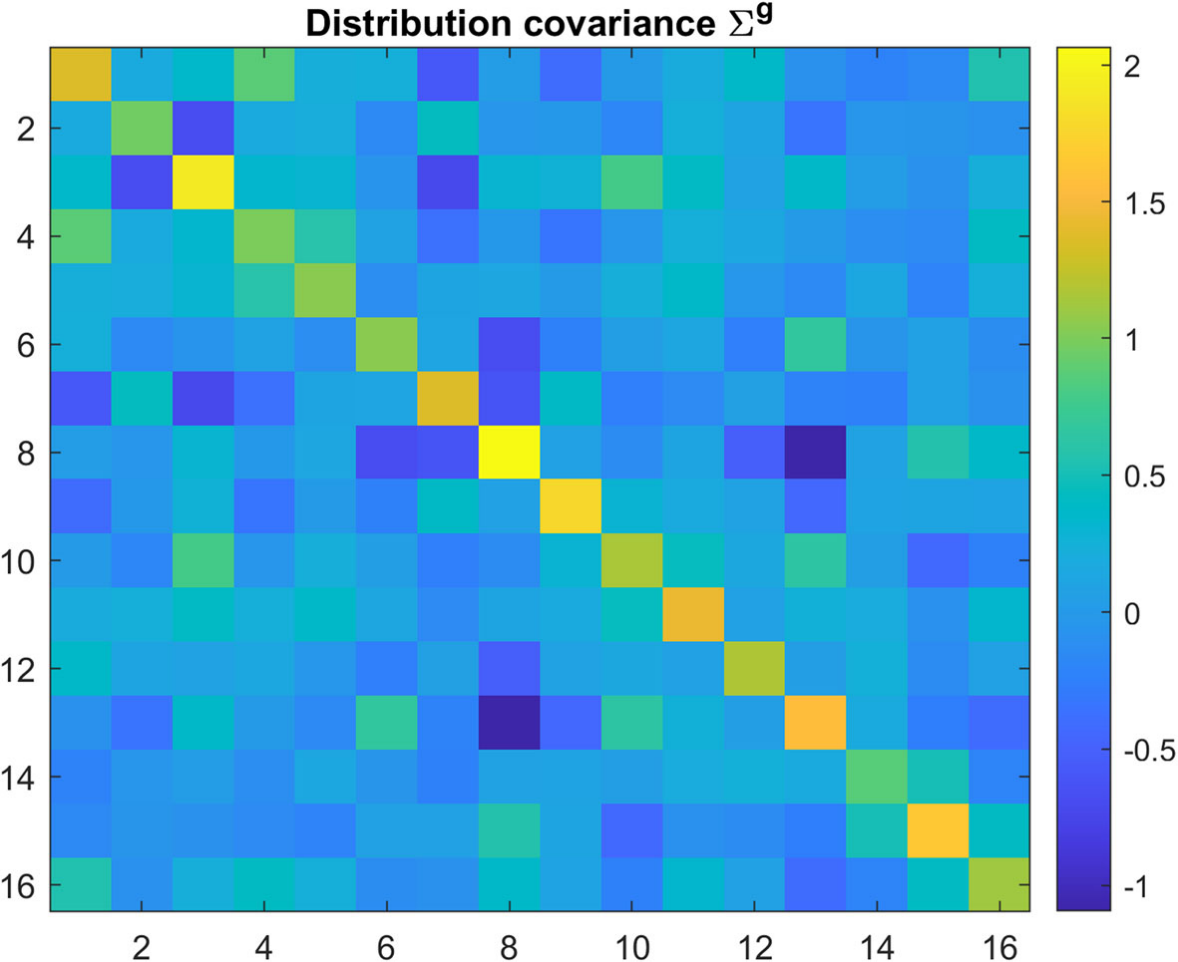
Generated Distribution Covariance: Covariance matrix of the principle vector *w* used to synthesize more general data

To generalize from the data found in the *Moral Machine Experiment*, an abstract dilemma in the same decision framework was also simulated and tested. This dilemma arbitrarily featured length 16 parameter vectors *θ*. To keep the dilemma as general as possible, the decision process is still modeled as an evaluation of utility *U*(*θ*), but no overlap between these parameters is assumed (i.e. the transform *A* is simply the identity matrix without loss of generality). The randomly generated *w*^*g*^,*Σ*^*g*^, and *κ* values used for this simulation are included in Figs. 7, 8 and Table 2.

**Table 2:** *κ* values generated for five non-Gaussian distributions used to synthesize more general data

∣*k*∣=	0.5	1	1.5	2	2.5
Abstract value					
1	-0.96	1.35	1.38	-2.52	3.79
2	0.64	1.45	-1.89	-1.9	0.93
3	-0.54	0.42	1.9	2.24	1.65
4	-0.33	0.65	1.27	-2.79	-1.5
5	0.08	0.89	-1.18	2.59	1.76
6	-0.41	-0.32	-0.82	2.8	-2.95
7	-0.06	-1.41	-1.7	1.01	2.65
8	0.7	1.4	1.97	-1.6	-2.12
9	-0.51	0.95	-0.65	-2.97	1.5
10	0.15	1.15	-1.35	1.05	2.67
11	0.87	-1.3	-0.8	-2.55	3.32
12	0.8	-1.07	1.92	2.1	-3.85
13	0.96	0.34	1.77	-0.92	-0.41
14	-0.28	1.4	1.7	2.1	3.92
15	0.49	-0.49	-1.96	1.91	3.95
16	0.23	-1.34	-1.54	-1.13	-2.98

## Results and discussions

### Training of DL model

**Fig. 9:**
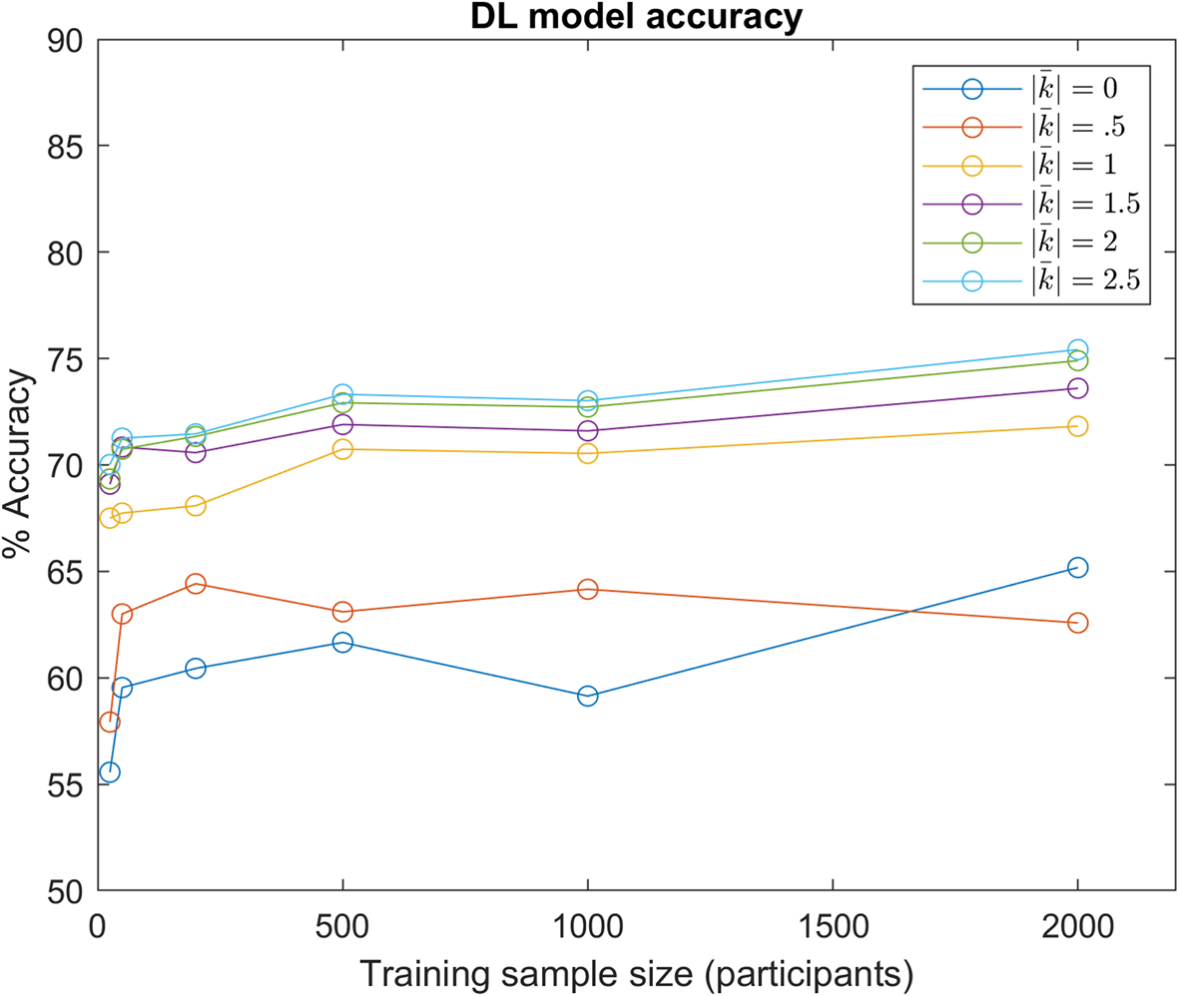
DL Accuracy: DL model predictive accuracy with different sample sizes and underlying distributions of *w*. Various distributions for *w* are denoted by the approximate average of absolute shape factors *k* ($\left |\bar {k}\right |=0$ denotes the Gaussian distribution)

Figure 9 illustrates the accuracy of the DL model with various training sample sizes and underlying distributions of *w*. The approximate average of the absolute value of the shape factor *k* correlates with the average skewness of the underlying distribution denoted as $\bar {\left |k\right |}$, the average skewness of underlying marginal distributions.

Unsurprisingly, the predictive accuracy increases with greater training samples, as the model is given a greater sample size to learn the distribution (although a size of 1,000 participants appears to perform slightly worse in certain instances). What is slightly unanticipated is the significant performance increase with increasing *w* distribution skewness. Despite all distributions sharing the same mean and covariance, the network can better predict decisions from participants sampled from a more skewed distribution. This is likely because an unskewed Gaussian distribution maximizes entropy for a fixed variance. That is, distribution entropy may decrease with increasing skew.

**Fig. 10:**
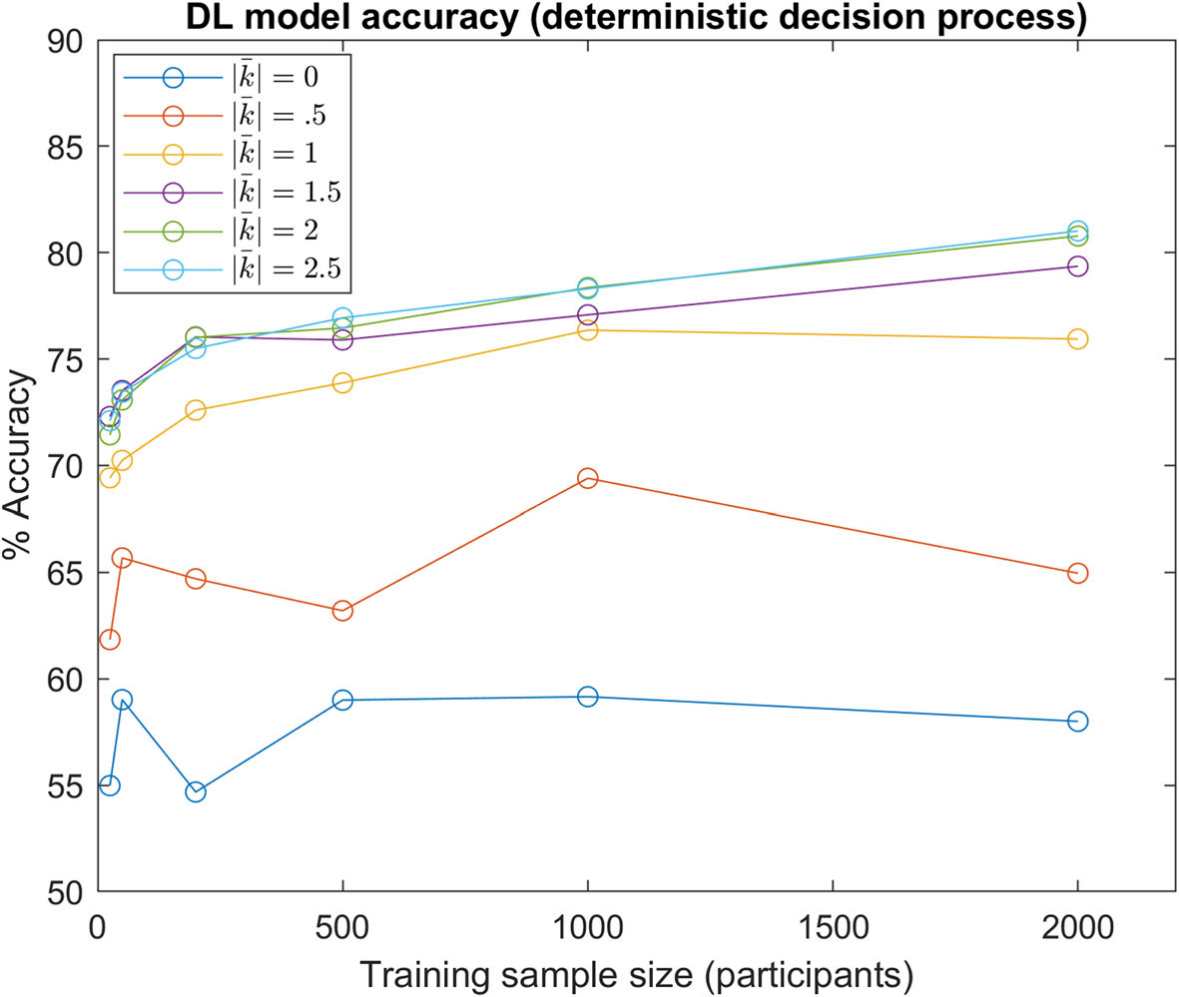
DL Accuracy (Deterministic Decision Process): DL model predictive accuracy with different sample sizes and underlying distributions of *w*. Various distributions for *w* are denoted by the approximate average of absolute shape factors *k* ($\left |\bar {k}\right |=0$ denotes the Gaussian distribution) In this test, decisions were simulated with the deterministic version of Eq. 2

Within the results above, all model instances are performance-limited by the stochastic nature of the decision making process in Eq. 2: even if a model implicitly estimates moral values perfectly, the model can still only predict the maximum likelihood decision, and not the decision itself. To observe trends without this source of randomness, models were also tested with data simulated from a deterministic version of Eq. 2, where the maximum likelihood decision was always selected (results shown in Fig. 10). It can be seen that this change generally amplifies differences in performance between the distributions.

Interestingly, fine-tuning the baseline DL model with 8 individual-specific samples did not significantly increase the model’s performance. As such, the predictions of the DL model are purely based on group observations, and does not account for individual differences. This suggests that in this instance, 8 individual-specific questions is insufficient to benefit the predictive accuracy of the DL. It is hypothesized that as in-group variances increase, the need for effectively accounting for individual differences would increase.

### Comparison of model performances

**Fig. 11:**
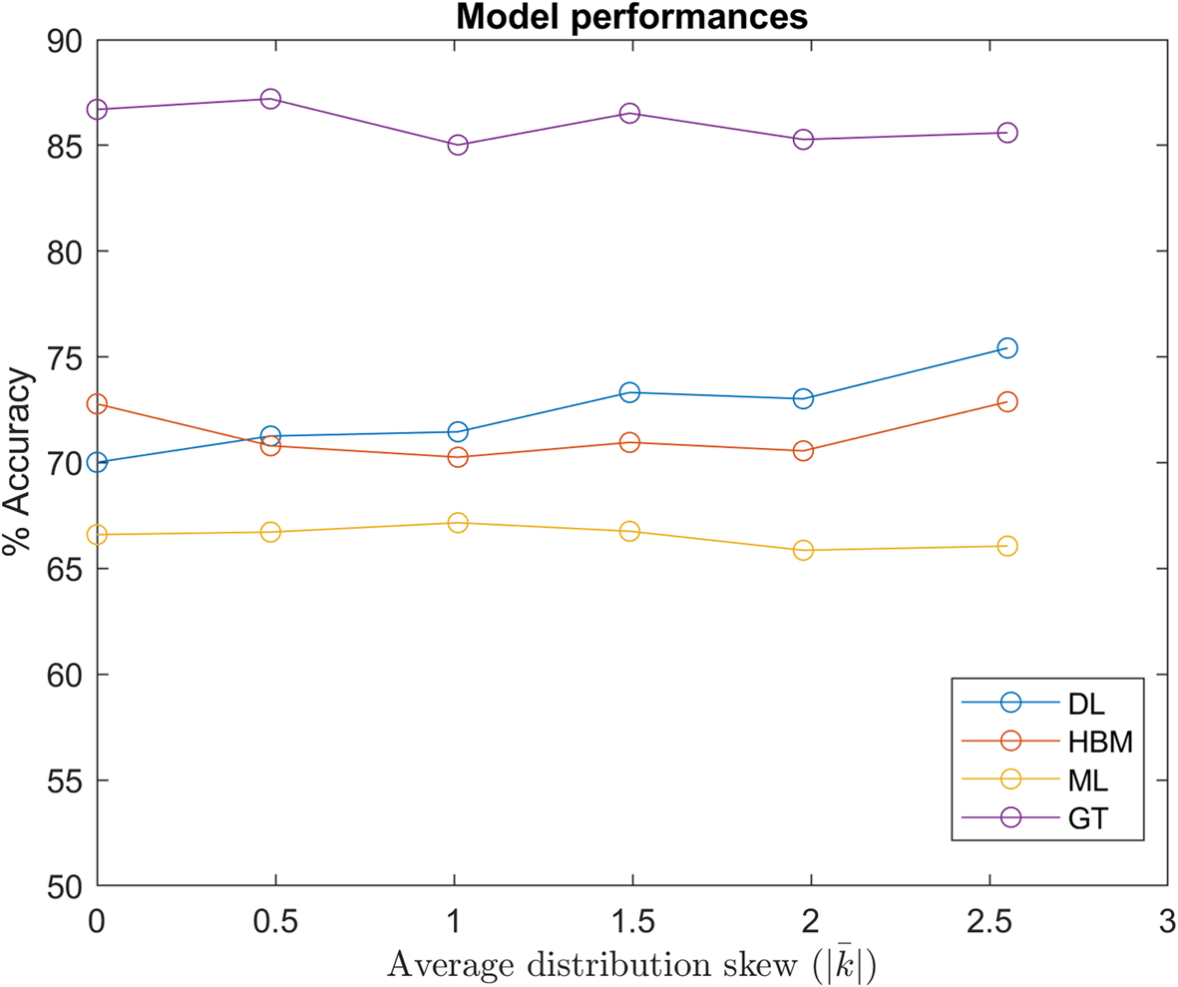
Model Performances: Comparison of model performances (DL trained with 2,000 participants involved) over datasets with various underlying distributions of *w*, denoted by the approximate average of absolute shape factors *k* ($\left |\bar {k}\right |=0$ denotes the Gaussian distribution). ‘GT’ denotes the predictive model in which the exact *w* for each participant is known

**Fig. 12:**
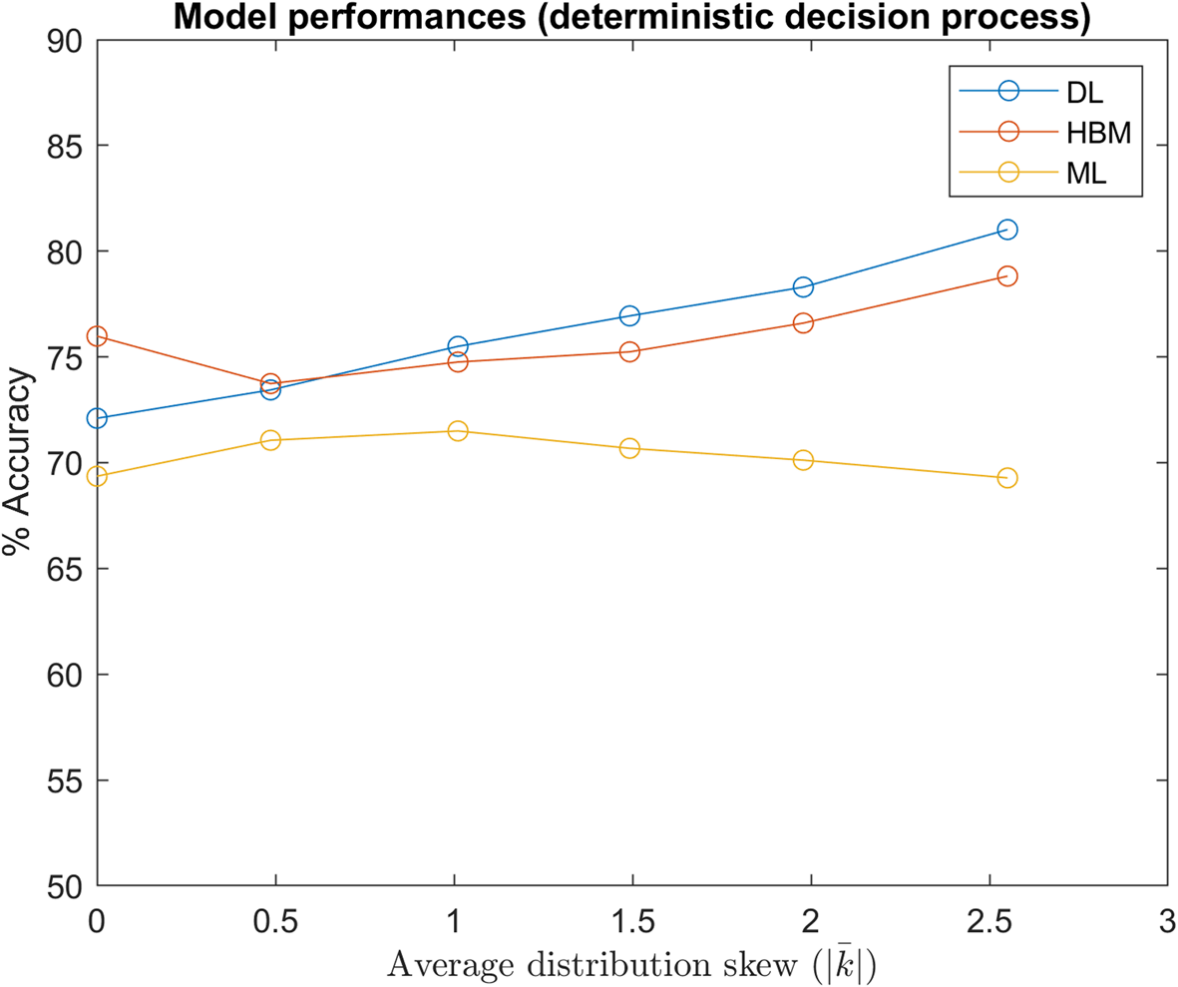
Model Performances (Deterministic Decision Process): Comparison of the model performances (DL trained with 2,000 participants involved) over datasets with various underlying distributions of *w*, denoted by the approximate average of absolute shape factors *k* ($\left |\bar {k}\right |=0$ denotes the Gaussian distribution). In this test, decisions were simulated with the deterministic version of Eq. 2

Figure 11 compares of model performances over different distributions. The ‘ground truth’ (GT) model is a predictive model where individual values *w* are exactly known. Thus, GT represents an upper limit the in predictive performance, limited only by the inherent randomness in Eq. 2. By comparison, Fig. 12 illustrates model performances when decisions are based on the maximum likelihood of Eq. 2 (GT accuracy = 100%).

The results shown in Figs. 11 and 12 indicate that a deep learning based model outperforms a hierarchical Bayesian model when the underlying distribution of *w* is skewed. On one hand, this is unsurprising, since the HBM’s prior assumed a normally distributed *w*. However, it is also worth noting that DL was able to achieve this accuracy without individual-specific data. Of course, we do not suggest that individual-specific data is unimportant when modeling moral principles. It is logical that individually-specific data would become increasingly important for accurate modeling as in-group variance increases. Indeed, we believe this implies a robust ability for neural networks to learn underlying group trends and decision processes, given enough training data. This ability is crucial, as the true distributions of population moral values, as well as how they affect moral decisions, are unknown.

In contrast to the DL model, the ML model’s performance was mostly invariant of the underlying distribution of *w* because it based predictions only on limited, individual-specific data, without any prior assumption. This implies that the information used by the DL and ML models are largely disjoint. As such, a model that effectively leverages population information via deep learning and limited individual-specific data via a maximum likelihood could be superior.

It is also worth noting that the HBM still outperformed the ML model in all instances, indicating that in this case a normal prior is still superior to no prior, as this assumption is still close to the actual underlying distribution. In fact, an increasing trend in the HBM accuracy beyond $\bar {\left |k\right |}=0.5$ suggests performance gains due to a lower entropy in more skewed distributions counteracted performance losses from an incorrect prior assumption.

### Further evaluations with generalized data

**Fig. 13:**
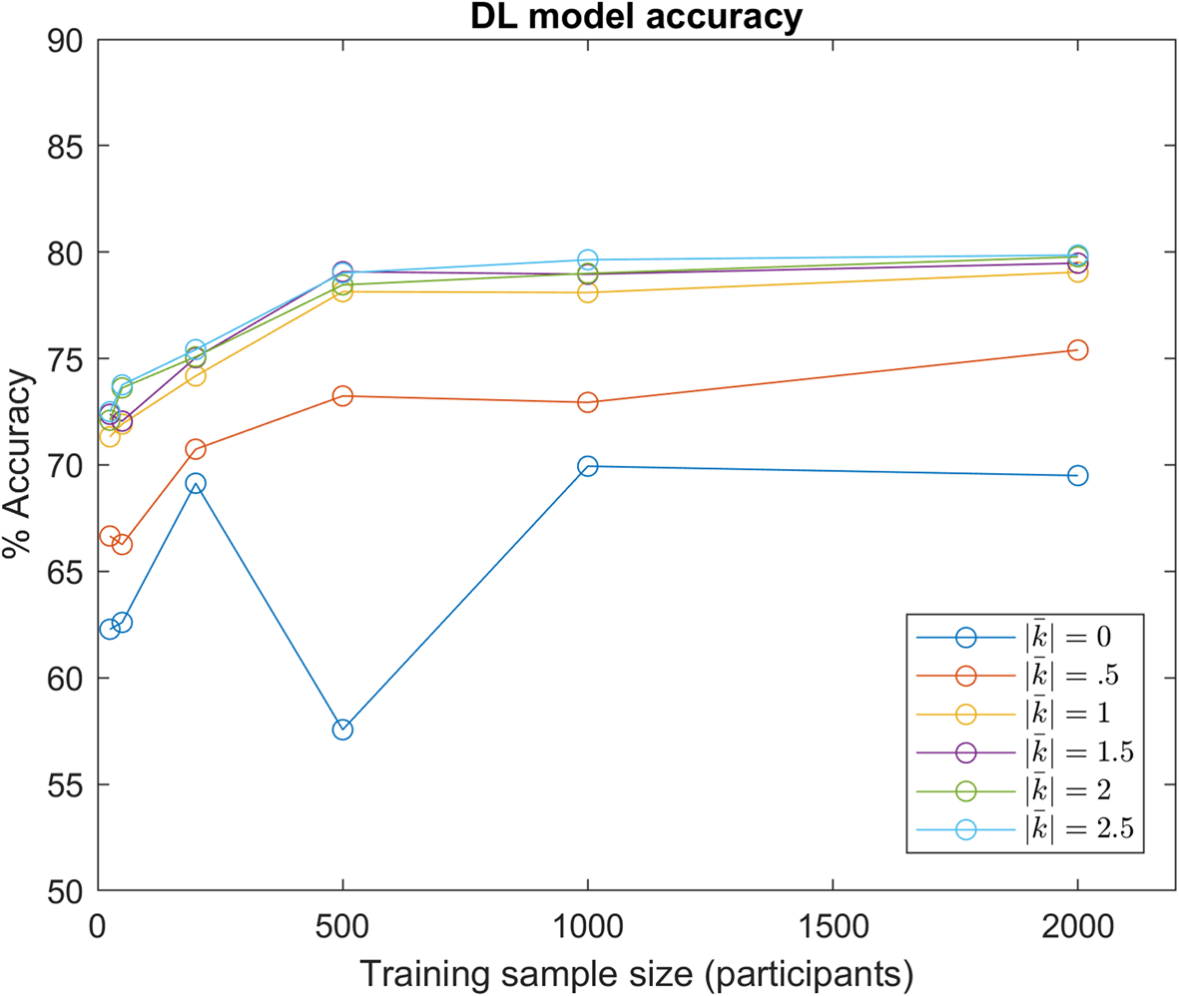
DL Model Accuracy (Generated): DL model predictive accuracy with different sample sizes and underlying distributions of *w*, applied to the generalized, abstract data. Various distributions for *w* are denoted by the approximate average of absolute shape factors *k* ($\left |\bar {k}\right |=0$ denotes the Gaussian distribution)

**Fig. 14:**
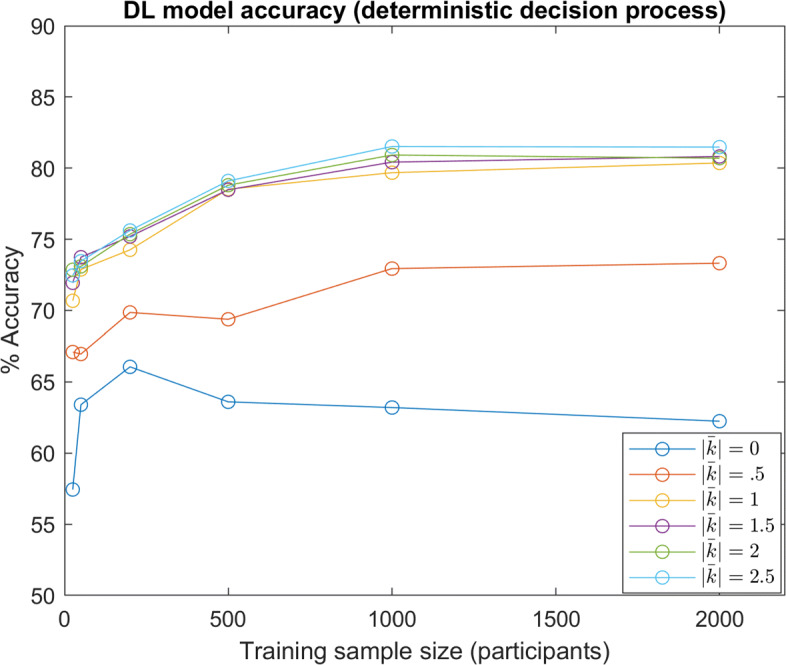
DL Model Accuracy (Generated; Deterministic Process): DL model predictive accuracy with different sample sizes and underlying distributions of *w*, applied to the generalized, abstract data. Various distributions for *w* are denoted by the approximate average of absolute shape factors *k* ($\left |\bar {k}\right |=0$ denotes the Gaussian distribution) In this test, decisions were simulated with deterministic version of Eq. 2

**Fig. 15:**
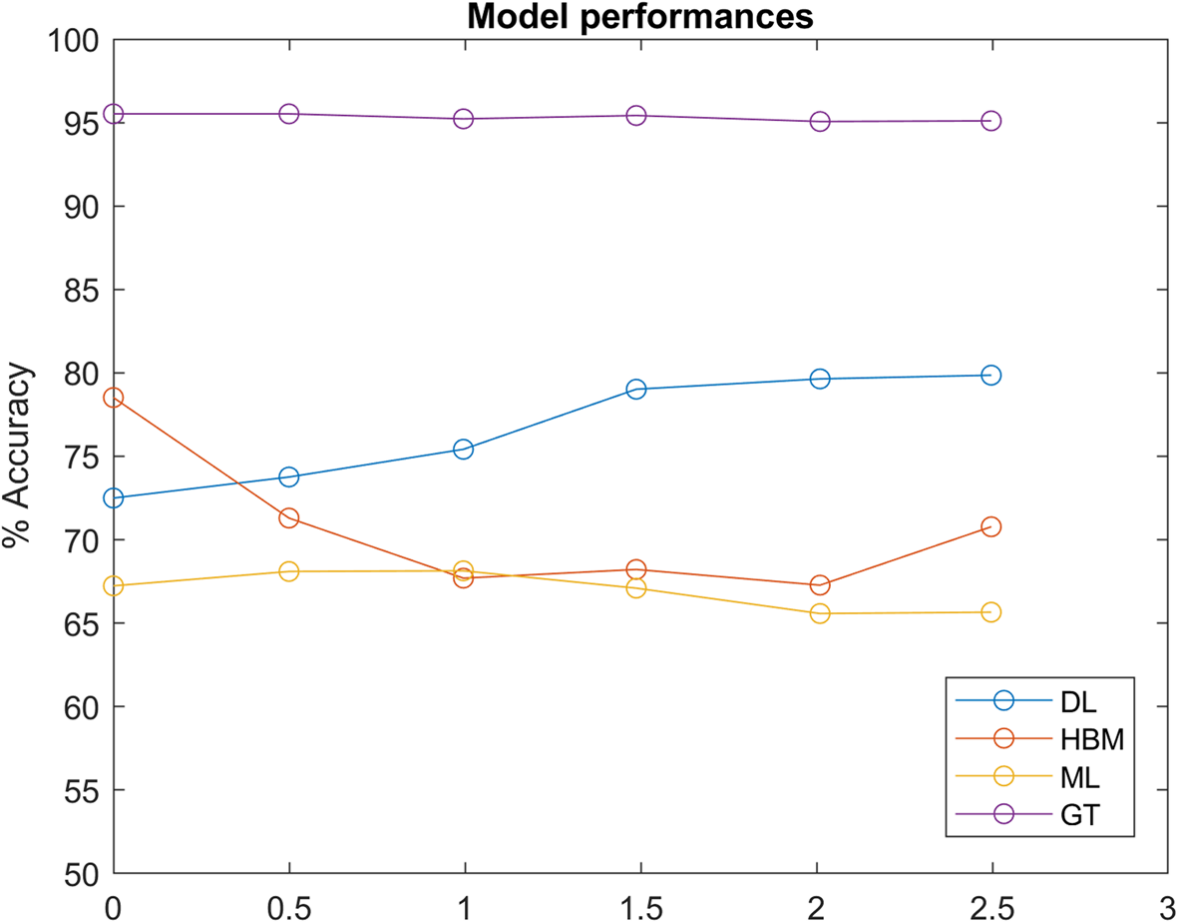
Model Performances (Generated): Comparison of model performances (DL trained with 2,000 participants involved) over datasets with various underlying distributions of *w*, denoted by the approximate average of absolute shape factors *k* ($\left |\bar {k}\right |=0$ denotes the Gaussian distribution), as applied to the generalized, abstract data. ‘GT’ is the predictive model in which the exact *w* for each test participant is known

**Fig. 16:**
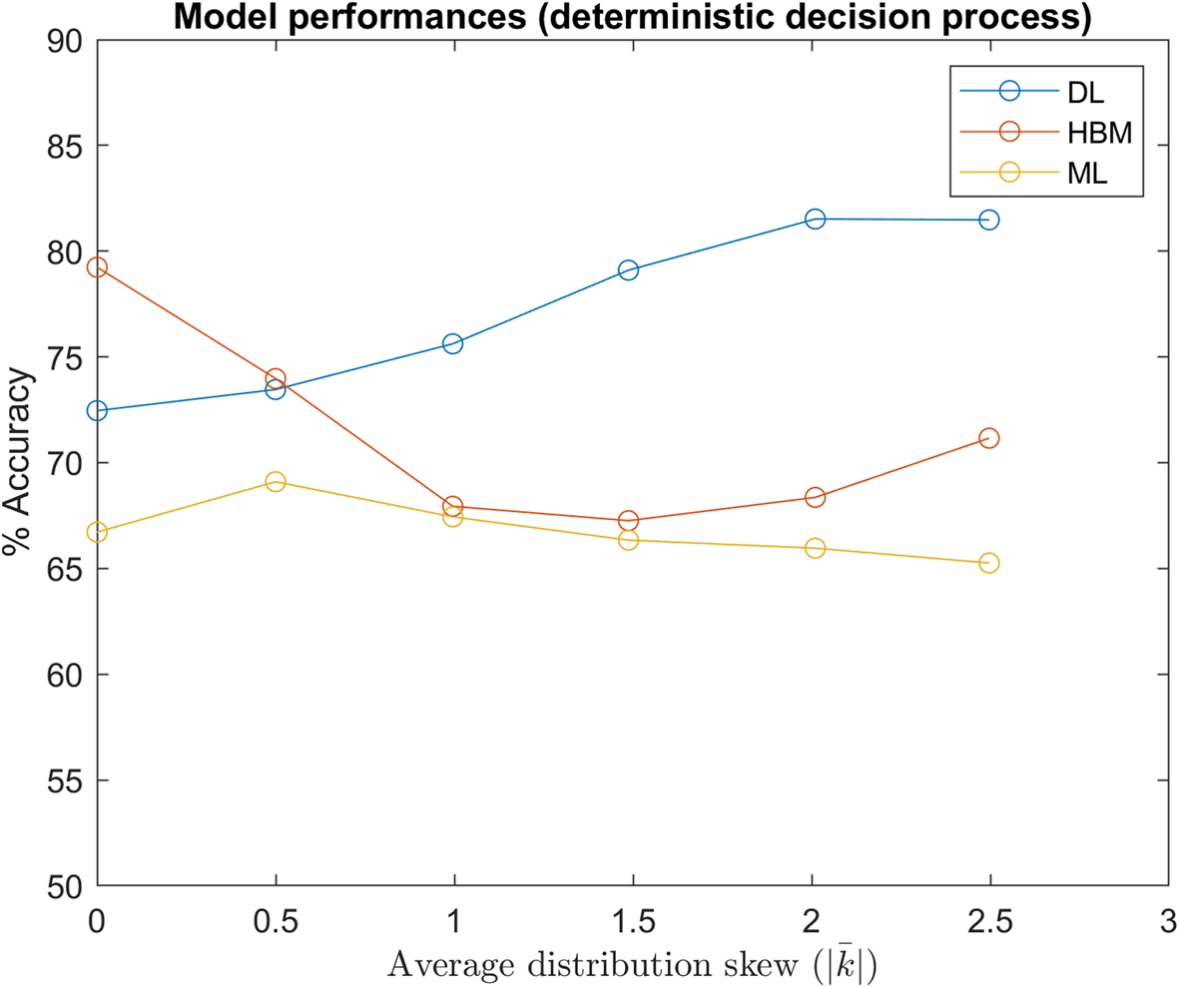
Model Performances (Generated; Deterministic Process): Comparison of model performances (DL trained with 2,000 participants involved) over datasets with various underlying distributions of *w*, denoted by the approximate average of absolute shape factors *k* ($\left |\bar {k}\right |=0$ denotes the Gaussian distribution), as applied to the generalized, abstract data. In this test, decisions were simulated with the deterministic version of Eq. 2

Models tested with the randomly generated abstract data were also analyzed, assuming both a random and deterministic decision process. Figures 13 and 14 plot the DL predictive accuracy over training size with the abstract data, while Figs. 15 and 16 compare the performances for each model type with this dataset. Overall, the trends seen in these experiments are consistent with the results seen in the specific, autonomous vehicle scenario. This demonstrates that these findings are not unique to the specific population and scenario parameters found in the autonomous vehicle instance.

## Conclusion

Overall, we have demonstrated that a deep learning based model can be effective in learning both moral values and making moral decisions in a data-driven fashion. Furthermore, the deep learning model is highly adaptive to training examples, requiring no assumption regarding the distribution of moral values in a population, or the decision process as a function of moral values. Given sufficient training data, this deep learning approach has a distinct advantage, since underlying moral value distributions and decision processes are generally unknown. Based on our initial findings, we are confident that this work inspires much needed confidence in deep neural networks for creating moral agents, given the robust results that deep neural networks show in this example. We are also confident that the parameterization of a moral dilemma used in this experiment can be applied to other, more complex moral scenarios and network architectures.

Future work on machine learning of individual moral decision making should apply the basic method to deep learning with morality shown here to more complicated models involving multiple AI agents, such as the approach suggested in [4] Other research could also leverage a deep learning model combined with a maximum likelihood component to better extract both group trends and individual specific information from limited data, or could train deep neural networks to weight both moral and legal considerations, which is an issue explored in [11]. We are particularly interested in two important applications of this proposed deep learning approach for decision making: 1) democratized re-opening decisions in a pandemic situation, and 2) AI-aided consent processes in healthcare. Further research opportunities are numerous.

## Data Availability

The datasets generated and/or analysed during the current study are available in the WANG-AXIS / Modeling-of-Moral-Decisions-with-Deep-Learning repository, https://github.com/WANG-AXIS/Modeling-of-Moral-Decisions-with-Deep-Learning.

## Abbreviations

AI: Artificial intelligence
DL: Deep learning
PDF: Probability density function
HB: Hierarchical Bayesian
ML: Maximum likelihood
GT: Ground truth
